# Framework for Bidirectional Knowledge-Based Maintenance of Wind Turbines

**DOI:** 10.1155/2022/1020400

**Published:** 2022-12-02

**Authors:** Javier Vives, Juan Palaci, Janverly Heart

**Affiliations:** ^1^Department of Systems Engineering and Automation, University Polytechnic of Valencia, Camino de Vera S/N, Valencia 46022, Spain; ^2^Red Engineering Technology Limited, 23 Radcliffe St, Wolverton, Milton Keynes, UK

## Abstract

Artificial intelligence (AI) techniques, such as machine learning (ML), are being developed and applied for the monitoring, tracking, and fault diagnosis of wind turbines. Current prediction systems are largely limited by their inherent disadvantages for wind turbines. For example, frequency or vibration analysis simulations at a part scale require a great deal of computational power and take considerable time, an aspect that can be essential and expensive in the case of a breakdown, especially if it is offshore. An integrated digital framework for wind turbine maintenance is proposed in this study. With this framework, predictions can be made both forward and backward, breaking down barriers between process variables and key attributes. Prediction accuracy in both directions is enhanced by process knowledge. An analysis of the complicated relationships between process parameters and process attributes is demonstrated in a case study based on a wind turbine prototype. Due to the harsh environments in which wind turbines operate, the proposed method should be very useful for supervising and diagnosing faults.

## 1. Introduction

In the last few years, the increase in energy consumption and the powerful potential of artificial intelligence algorithms have changed the current supervision and fault diagnosis of the industrial process. In the last 5 years, the wind power infrastructure has increased wind production by about 80% around the world [1]. Researchers have developed new techniques to maintain wind power, combining the traditional methods of monitoring and supervision with machine learning (ML) techniques. In the literature [2, 3], there are several studies combining the methodology of ML for the maintenance of wind turbines. Several industries are using predictive maintenance because of its unique processing characteristics, such as aerospace, oil and gas, nuclear, automotive, and shipbuilding [3]. It is estimated that the size of the market for ML in the industrial sector will exceed USD 2000 million in the near future, according to a market survey [4]. One wind turbine is built with thousands of different components that must be perfectly matched and synchronized to work and achieve the best possible performance. One of the most critical components that can fail in a wind turbine are the bearings, blades, and gears. Offshore wind farms must detect and diagnose faults early if they are to be stopped in case of problems, especially those that have high repair and maintenance costs, especially if they are offshore [5]. As well as minimizing downtime and defect costs, maintenance activities must be managed efficiently. By applying algorithms designed to anticipate and prevent problems, we developed a prototype that detects, supervises, and anticipates failures in contrast to existing systems. Currently, the monitoring and prediction systems for possible failures in wind turbines focus on vibrations, shaft speed, noise, and even overheating of some components. Using digital and artificial intelligence technologies, such as machine learning, to create a fast and accurate prediction system for wind turbines is an essential research topic [6]. Digital solutions and machine learning for rotatory machines have been the subject of many research endeavours, such as prediction and control of the fast and slow axes [7], bearing defect detection [8, 9], or system vibrations [10, 11].

ML is being applied to wind turbines in several recent ways. Sun et al. [12] adopted a neural network as the main method for the yaw angle prediction. To forecast the process parameters precisely according to the desired blade yaw angles, the forward neural network model is needed to work with a backward prediction strategy. Two output process parameters were obtained using only two input features of wind speed and air density in the reverse model. Jiménez et al. [13] designed and developed an ML algorithm for the real-time maintenance and prediction of a possible blade failure, comparing different supervised ML methods such as, for example, decision trees, discriminant analysis, support vector machines (SVM), or nearest neighbours (KNN). Compared to the traditional optimisation approach (ML), bidirectional predictions were considered much more efficient in their research. It is possible to dramatically reduce maintenance costs by adopting this cutting-edge technology.

This paper presents a knowledge-based ML modelling framework that enables two directions of prediction. In the forward direction, the basic process parameters pertinent to a specific process, such as temperature and axis speed, are applied as the initial model inputs, while knowledge-based process factors are added as the advanced model inputs. Using training data obtained from previous experiments, knowledge-based ML is then applied to predict process attributes. The process parameters are derived from the knowledge-based features after the process factors are predicted based on the requirements of the process attributes. For understanding, analysing, designing, and optimizing wind turbine maintenance processes, forward and backward predictions are important. An evaluation of the suggested approach is also presented in this paper through a case study. This paper is structured as follows: in Section 2, the knowledge-based ML modelling framework is introduced.  Section 3 displays the prototype and the variables that are monitored via forward and backward modelling. The results, performance, and predictions of the algorithm are shown in Section 4.  Section 5 concludes the research, highlighting the main conclusion.

## 2. Methodology

**Figure 1:**
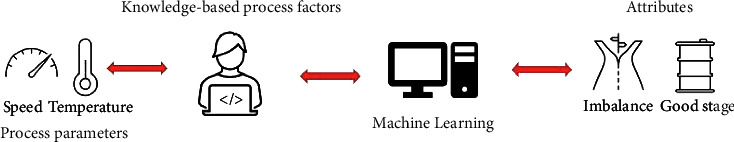
Bi-directional modelling framework based on knowledge.

ML models and process factors are combined to propose a bidirectional modelling approach that predicts the attributes of the process in this section, such as imbalance and good stage bearings, and predicts basic process parameters, such as the temperature and speed of the wind turbine axis.  Figure 1 illustrates the framework. This framework defines ML models as being both the input and output of basic process parameters and key process attributes. To enhance the performance and efficiency of ML models, knowledge-based factors are introduced.

### 2.1. Process Parameters

As the most important input for monitoring a wind turbine process, the basic process parameters always need to be defined carefully to achieve the required part performance. Depending on the wind turbine scale and location, various parameters are defined and used. For instance, temperature and rotor speed are two of the main process parameters for a wind turbine. There are also some other basic process parameters, like wind speed, wind direction, and blade radius. When their effects are revealed, these parameters can also be called basic process parameters. In this document, these basic process parameters are represented as *A*_*b*_ (*n* is the number of process parameters).

(1) $$\begin{array}{c} A_{b}=\left[A_{1},A_{2},A_{3},\ldots ,A_{n}\right]\text{.} \end{array}$$

### 2.2. Process Factors Based on Knowledge

Research has shown that the process attributes are generally determined by different basic process parameters that are fed directly into the modelling process, as described in the introduction. Different combinations of basic process parameters can, however, produce the same process attributes. For bidirectional modelling, the basic process parameters need to have a unique relationship. To link the process attributes directly, knowledge-based process factors were utilized in this study. The process factors can be determined according to the basic parameters of the process as well as their physical mechanisms. As a result, the data-driven model will be less redundant when the ML model is applied.

As a result of applying the process factors, the fundamental relationship can be embedded in the model, allowing for more efficient data analysis and a more generic solution for different types of wind turbines. As an example, several factors can be used, such as air or density temperature, to determine the imbalance or good stage variables. Based on the prior knowledge and basic parameters, new features can be generated, which are donated as *A*_*k*_.

(2) $$\begin{array}{c} A_{k}=T_{k}\left(A_{b}\right) \end{array}$$

where *T*_*k*_(*∗*) represents the transfer function based on the physical mechanism for the axis turn.

### 2.3. Process Attributes

Many of the attributes of a process are determined by digital modelling or simulation, but some can be measured directly from the real-time monitoring of the process, which can be represented as *X*_*at*_. This type of digital model is typically computationally demanding and requires experimental data as input. As a result, we can express the process attribute as follows:

(3) $$\begin{array}{c} X_{at}=T_{d}\left(D_{ex}\right)\text{,} \end{array}$$

where *T*_*d*_(*∗*) represents the function of the digital model, and *D*_*ex*_ is the data collected from experiments.

### 2.4. Bidirectional Prediction with Machine Learning

A neural network is the main machine learning (ML) technology used in this research, while other algorithms are compared in the case study. Data properties and problems can dictate the type of ANN [14] to be used. Typically, recurrent neural networks (RNNs) are used for processing real-time monitoring data, whereas convolutional neural networks (CNNs) are used for processing image data. During this research, the model is both input and output by using parameters, factors, and attributes. Using a forward model, we can say,

(4) $$\begin{array}{c} X_{at}=T_{ML}\left(T_{k}\left(A_{b}\right)\right)=T_{d}\left(D_{ex}\right), \end{array}$$

where *T*_*ML*_(*∗*) is the ML algorithm. According to the backward model,

(5) $$\begin{array}{c} A_{b}=T_{k}^{-1}\left(T_{ML}\left(X_{at}\right)\right)\text{.} \end{array}$$

## 3. Case Study

An evaluation of the proposed modelling framework is demonstrated in this section. Several previous experiments and simulations on a wind turbine prototype have generated a fault variable dataset that can be used to read the process attributes (imbalance or good stage). During the experiments, the design of the experimental method was used to minimise the number of samples required. The implemented method was according to [15]. A wide range of process parameters was adopted, such as air temperature (10–25°C), wind speed (5–20 km/s), and air density (1.225 km/m^3^).

### 3.1. Temperature Model

The temperature sensors are used to detect overheating in the components and determine which faults they correspond to, based on the studies carried out on the rest of the components. In this study, the temperature above the shaft bearing is monitored. The selected temperature sensors are positive temperature coefficient sensors, that is, PTC-type sensors, specifically the PT-100. For the temperature sensors, two NI 9217 modules [16] have been used. The NI 9217 RTD Analog Input Module has 4 channels and 24-bit resolution for 100 Ω RTD measurements. The NI 9217 can be configured for two different sampling modes. Both the NI 9201 and NI 9217 boards mount on the NI cDAQ-9172 module [17], which is an 8-slot compact DAQ chassis that can support up to eight I/O modules. It operates from 11 to 30 VDC and includes an AC/DC power adapter.

### 3.2. Speed Model

**Figure 2:**
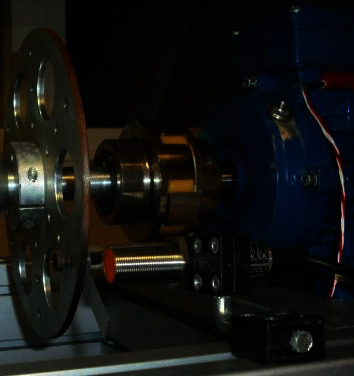
Positioning of the speed sensors on the prototype.

The measurement of the speed is important to know the relative movement of the slow and fast axes and thus check the correct operation of the system, as well as possible failures, by comparing this signal with others of the acquired ones. Likewise, the data will be used to check the status of the machine in case it is stopped. For the speed measurements, two inductive proximity sensors were selected, specifically the IG5594 [18], which were installed on each of the axes, one for the slow axis and one for the fast axis, as you can see in Figure 2. By their nature, inductive speed sensors work with pulsed signals, delivering a high level when the sensor detects metal. Both sensors connect to the NI 9201 module on top of the NI cDAQ-9172.

### 3.3. Modelling Bi-Directionally with Machine Learning

We propose a bidirectional model, which consists of forward and backward prediction models. An ML model for forward and backward prediction embeds process knowledge using two process factors. Mainly, ANNs were used for ML modelling. It was possible to make three predictions using two different neural networks using both forward and backward modelling approaches.

#### 3.3.1. Forward Modelling

**Figure 3:**
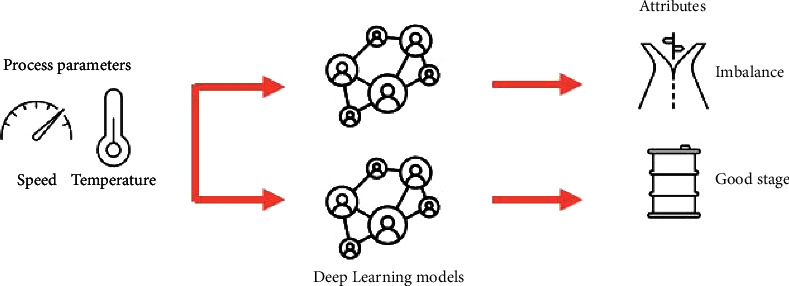
Modelling variables using forward process knowledge-based machine learning.

In Figure 3, we see how the forward model is structured. A basic process parameter, such as wind speed and temperature, is the first input, and a good stage or imbalance variable is the target. A neural network structure optimized for each neural network was designed separately.

#### 3.3.2. Backward Modelling

**Figure 4:**
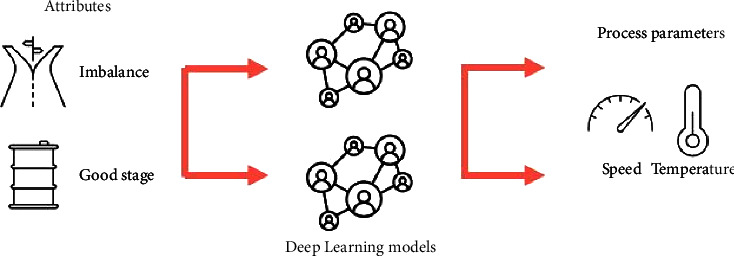
Modelling variables using backward process knowledge-based machine learning.

As shown in Figure 4, the target parameters of the backward model are the basic process parameters, while the imbalance or good stage variables are the input variables. In the first step, neural networks are used to predict the process factors. Using the relationship between the process parameters and the process, the basic parameter values are calculated.

## 4. Results

**Figure 5:**
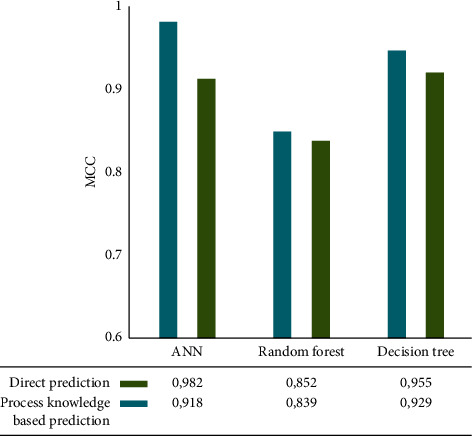
Modelling performance for good stage prediction. Forward model.

We evaluated the proposed modelling approach using a model correlation coefficient (MCC). Additionally, two other ML algorithms, namely, decision trees and random forests, were compared with the results obtained from the proposed approach based on ANN. All results from ANN and other ML algorithms, as well as those without knowledge-based process factors, are compared to the results from ANN and other ML algorithms without process factors. A good stage and imbalance were predicted by forward modelling, as described in Section 3.3.1.  Figure 5 shows the predictions for good stages. Direct prediction and process factors produced the highest MCC, 0.98, according to the proposed approach. Without incorporating process factors, random forest modelling made the least accurate predictions. The MCC of all ML algorithms was also improved by about 5% when process factors were used as enhanced inputs.

**Figure 6:**
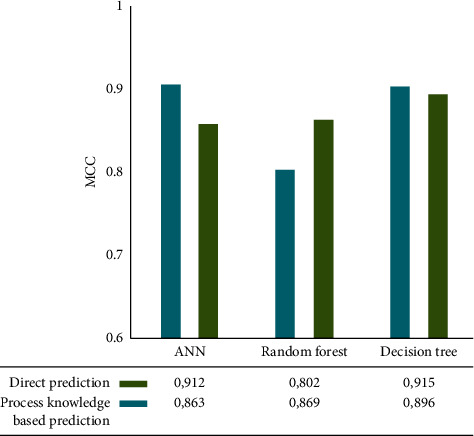
Modelling performance for imbalance prediction. Forward model.

According to Figure 6, the prediction results of imbalance using ANN, random forest, and decision tree show similar trends in comparison with the prediction results of the good stage. Direct prediction with and without process factors obtained the highest MCC, in this case, 0.92 MCC. A decision tree algorithm was used to calculate this value. Using both the random forest algorithm and direct prediction, the random forest algorithm provided the lowest MCC (0.802). Most ML models do not show a significant improvement in prediction efficiency when knowledge-based process factors are considered.

**Figure 7:**
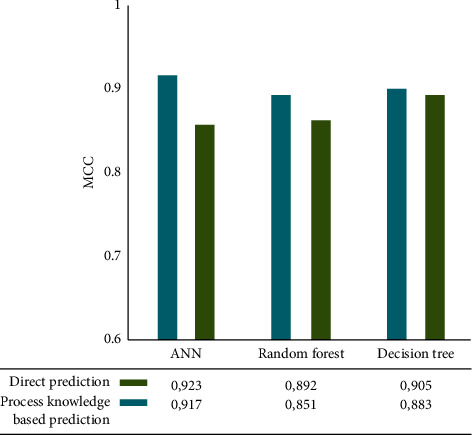
Modelling performance for good stage prediction. Backward model.

Temperature and speed are the primary targets of the backward model. The prediction results for temperature are shown in Figure 7. With an MCC of 0.93, ANN is more accurate than other ML technologies. Based and direct predictions are very similar to the ANN methodology. Our lowest MCC (8.85) was obtained using the process knowledge-based prediction algorithm.

**Figure 8:**
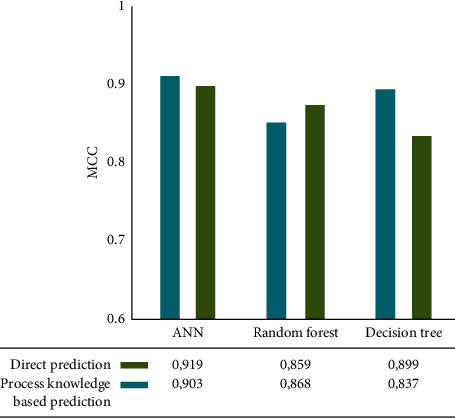
Modelling performance for imbalance prediction. Backward model.

Figure 8 shows the prediction results of speed. The ANN is also the most accurate in direct prediction, with an MCC of 0.92, similar to the previous variable. For other ML technologies in direct prediction, using the decision tree algorithm, we obtained a 0.89 MCC, while using a random forest, we scored the lowest MCC (0.86). If we compare the results with the based predictions, the results are very similar; just in this case, the lowest MCC (0.837) is obtained by the decision tree.

When the process of factor-based prediction and direct prediction in both models (forward and backward) using the ANN algorithm were compared, this the methodology produced the best results. The ANN-based modelling has improved and shown better results than the other ML technologies by around 7%. In conclusion, ANNs supported by process factors are more effective than other ML algorithms when compared with other algorithms. The ANN is less sensitive to different targets and is better at adapting to new input features.

## 5. Conclusions

Supervisory and fault diagnosis of wind turbines can be accomplished through a data-driven approach. In this approach, physical and empirical relationships and process knowledge were integrated for bidirectional modelling. Based on a review of the state of the art, the proposed approach combines physics-based and data-driven models. As a result of both approaches proposed, a very good result has been achieved. As a result of understanding the fundamental relationship embedded in the physical mechanisms, the inputs and outputs of the proposed modelling are obtained. In this study, ANNs showed better results than ML algorithms. When basic process parameters are used as direct outputs for inverse analysis, the nonuniqueness issue generally arises, which is overcome by the knowledge-based, data-driven method. Due to its effectiveness, the methodology can be applied to other mechanical components of wind turbine prototypes, thus preventing the breakdown of other mechanical components. This prototype can be used to study, develop, and validate fault diagnosis and supervision techniques, with the possibility of replacing defective or worn parts with alternative components. High-performance wind turbines are equipped with prototype wind turbines that are used to test diagnostic algorithms. It also enables the algorithms to be verified, adjusted, and corrected, thus saving time and money.

## Data Availability

The data generated or analyzed during this study are included within the article.

## References

[1] Nejad A. R., Keller J., Guo Y. (2022). Wind turbine drivetrains: state-of-the-art technologies and future development trends. *Wind Energy Science*.

[2] Garan M., Tidriri K., Kovalenko I. (2022). A data-centric machine learning methodology: application on predictive maintenance of wind turbines. *Energies*.

[3] Leukel J., González J., Riekert M. (2021). Adoption of machine learning technology for failure prediction in industrial maintenance: a systematic review. *Journal of Manufacturing Systems*.

[4] Balachandar K., Jegadeeshwaran R. (2021). Friction stir welding tool condition monitoring using vibration signals and Random forest algorithm–A Machine learning approach. *Materials Today Proceedings*.

[5] Zhang L., Li Y., Xu W. (2022). Systematic analysis of performance and cost of two floating offshore wind turbines with significant interactions. *Applied Energy*.

[6] Badihi H., Zhang Y., Jiang B., Pillay P., Rakheja S. (2022). A comprehensive review on signal-based and model-based condition monitoring of wind turbines: fault Diagnosis and lifetime prognosis. *Proceedings of the IEEE*.

[7] Singh N., De Kooning J. D. M., Vandevelde L. (2022). Dynamic wake analysis of a wind turbine providing frequency support services. *IET Renewable Power Generation*.

[8] Yadav E., Chawla V. K. (2022). An explicit literature review on bearing materials and their defect detection techniques. *Materials Today Proceedings*.

[9] Zhou A., Ai B., Qu P., Shao W. (2021). Defect detection for highly reflective rotary surfaces: an overview. *Measurement Science and Technology*.

[10] Teng W., Ding X., Tang S., Xu J., Shi B., Liu Y. (2021). Vibration analysis for fault detection of wind turbine drivetrains—a comprehensive investigation. *Sensors*.

[11] Jahangiri V., Sun C., Kong F. (2021). Study on a 3D pounding pendulum TMD for mitigating bi-directional vibration of offshore wind turbines. *Engineering Structures*.

[12] Sun H., Qiu C., Lu L., Gao X., Chen J., Yang H. (2020). Wind turbine power modelling and optimization using artificial neural network with wind field experimental data. *Applied Energy*.

[13] Arcos Jiménez A., Zhang L., Gómez Muñoz C. Q., García Márquez F. P. (2020). Maintenance management based on machine learning and nonlinear features in wind turbines. *Renewable Energy*.

[14] Gawlikowski J., Tassi C. R. N., Ali M. (2021). A survey of uncertainty in deep neural networks. https://arxiv.org/abs/2107.03342.

[15] Vives J. (2022). Incorporating machine learning into vibration detection for wind turbines. *Modelling and Simulation in Engineering*.

[16] Jiménez-Castillo G., Muñoz-Rodríguez F. J., Rus-Casas C., Hernández J. C., Tina G. M. (2019). Monitoring PWM signals in stand-alone photovoltaic systems. *Measurement*.

[17] Qawqzeh Y. K., Bajahzar A. S., Jemmali M., Otoom M. M., Thaljaoui A. (2020). Classification of diabetes using photoplethysmogram (PPG) waveform analysis: logistic regression modeling. *BioMed Research International*.

[18] Quiles E., Garciia E., Cervera J., Vives J. (2019). Development of a test bench for wind turbine condition monitoring and fault diagnosis. *IEEE Latin America Transactions*.

